# Supplementary material for: Efficacy of Immune Checkpoint Inhibitor (ICI) Rechallenge in Advanced Melanoma Patients’ Responders to a First Course of ICI: A Multicenter National Retrospective Study of the French Group of Skin Cancers (Groupe de Cancérologie Cutanée, GCC)
Source: Cancers (Basel). 2023 Jul 10;15(14):3564. doi: 10.3390/cancers15143564 (PMC10377277; doi:10.3390/cancers15143564)
Supplement: Supplementary file 1 [file cancers-15-03564-s001.zip › cancers-2390307-supplementary.pdf]

Supplemental tables and figure:

Supplemental Table S1: Characteristics of the disease before first and second course (ICI rechallenge) of immune checkpoint inhibitor immunotherapy

	**First ICI**<br>**Total, *N* = 85, 100%**	**Second ICI**<br>**Total, *N* = 85, 100%**
**AJCC stage**		
IIIB (%)	4 (5)	0 (0)
IIIC (%)	15 (18)	7 (8)
**IIID (%)**	3 (3)	3 (4)
**IV (%)**	63 (74)	75 (88)
**Number of metastatic sites**		
≤3 (%)	40 (47)	48 (57)
> 3 (%)	45 (53)	37 (43)
**Brain metastasis**		
n (%)	16 (19)	22 (26)
**Elevated LDH level**		
No (%)	47 (55)	55 (65)
Yes (%)	12 (14)	11 (13)
Unknown (%)	26 (31)	19 (22)
**Corticosteroids (ICI rechallenge)**		9 (11)
> 10 mg/day (%)		6 (7)
≤ 10 mg/day (%)		3 (4)
**Immunotherapy (ICI)**		
Pembrolizumab (%)	41 (48)	44 (52)
Nivolumab (%)	27 (32)	35 (41)
Ipilimumab (%)	10 (12)	2 (2)
Ipilimumab + Nivolumab (%)	7 (8)	4 (5)
**Duration of ICI (months)**		
Median [range]	15 [1 – 51]	9 [1 – 72]
**Response to ICI**		
Complete response (%)	47 (55)	30 (35)
Partial response (%)	28 (33)	16 (19)
Stable disease (%)	10 (12)	18 (21)
Progression (%)	0 (0)	21 (25)
**Time to relapse (months)**		
Median [range]	14 [2 - 54]	11 [0.1 - 63]
**Time to best response to ICI (months)**		
Median [range]	11 [0.6 – 48]	3 [0.5 - 34]
**Toxicity**	43 (51)	23 (27)
Cutaneous (%)	14 (16)	8 (9)
Endocrinal (%)	14 (16)	4 (5)
Digestive (%)	7 (8)	6 (7)
Liver (%)	10 (12)	3 (4)
Respiratory (%)	3 (4)	2 (2)
Hematological (%)	1 (1)	2 (2)
Neuromuscular (%)	3 (4)	0 (0)
Renal	0 (0)	1 (1)
Rheumatological (%)	4 (5)	2 (2)
Cardiac (%)	1 (1)	0
**Grade of toxicity**		
Grade I (%)	10 (12)	6 (7)
Grade II (%)	15 (18)	7 (8)
Grade III (%)	17 (20)	8 (9)
Grade IV (%)	2 (2)	1 (1)
Grade V (%)	0	0
**Time to toxicity (months)**		
Median [range]	5 [0.6 – 43]	3 [0.4 – 36]

AJCC: American joint committee on cancer, ECOG: Eastern cooperative oncology group, ICI: Immune checkpoint inhibitor, LDH: Lactate dehydrogenase.

Supplemental Table S2: Univariate and multivariate analyses for factors associated with progression free survival during ICI rechallenge

	Univarious model			Multivarious model		
**Variable**	**HR**	**95% CI**	***P*-value**	**HR**	**95% CI**	***P*-value**
**Age (years)**	1.001	0.98, 1.024	0.958			
**Gender**			0.273			
Male	1	—				
Female	0.702	0.373, 1.321				
**ECOG performance status (ICI rechallenge)**			0.668			
0/1	1	—				
≥ 2	1.295	0.398, 4.218				
**Type of primary melanoma**			0.439			
Cutaneous	1	—				
Other	1.330	0.646, 2.737				
**BRAFV600 mutation**			0.600			
No	—	—				
Yes	1.203	0.603, 2.399				
**Response to 1st ICI**			0.511			
Complete response + Partial response	1	—				
Stable disease	1.318	0.578, 3.006				
**Duration of 1st ICI (months)**	1.022	0.99, 1.052	0.139	1.033	1.003, 1.063	0.031
**Time to relapse after 1st ICI (months)**			0.248			
≤ 6 months	1	—				
> 6 months	0.680	0.353, 1.309				
**Systemic treatment between the ICI courses**			0.008			0.016
No	1	—		1	—	
Yes	2.548	1.272, 5.104		2.382	1.173, 4.835	
**Local treatment between the ICI courses**			0.051			
No	1	—				
Yes	1.965	0.997 ; 3.872				
**Number of metastatic sites (ICI rechallenge)**			0.010			0.007
**> 3**	1	—		1	—	
**≤ 3**	0.448	0.243, 0.826		0.421	0.224, 0.793	
**Brain metastasis (ICI rechallenge)**			0.603			
No	1	—				
Yes	1.192	0.615, 2.312				
**Elevated LDH level (ICI rechallenge)**			0.312			
No	1	—				
Yes	1.717	0.730, 4.040				
Unknown	1.540	0.763, 3.109				
**Corticosteroids (ICI rechallenge)**			0.307			
No	1	—				
Yes	1.575	0.658, 3.765				
**2nd course of ICI (ICI rechallenge)**			0.385			
Rechallenge	1	—				
Escalation	1.528	0.364, 6.405				
Switch	0.473	0.143, 1.565				
**Toxicity (ICI rechallenge)**			0.163			
No	1	—				
Yes	0.599	0.292, 1.230				

CI: Confidence interval, ECOG: Eastern cooperative oncology group, ICI: Immune checkpoint inhibitor, LDH: Lactate dehydrogenase, OR: Odds ratio. Only factors which are significant are shown in multivariate analysis

Supplemental Table S3: Univariate and multivariate analyses for factors associated with overall survival during ICI rechallenge

	Univarious model			Multivarious model		
**Variable**	**HR**	**95% CI**	***P*-value**	**HR**	**95% CI**	***P*-value**
**Age (years)**	1.007	0.97, 1.040	0.677			
**Gender**			0.436			
Male	1	—				
Female	0.711	0.301, 1.679				
**Type of primary melanoma**			0.252			
Other	1	—				
Cutaneous	0.591	0.270, 3.077				
**BRAFV600 mutation**			0.971			
No	1	—				
Yes	0.980	0.362, 2.665				
**Response to 1st ICI**			0.267			
Complete response + Partial response	1	—				
Stable disease	1.770	0.646, 4.852				
**Duration of 1st ICI (months)**	1.019	0.98, 1.058	0.340			
**Time to relapse after 1st ICI (months)**			0.039			
≤ 6 months	1	—				
> 6 months	0.419	0.183, 0.96				
**Systemic treatment between the ICI courses**			0.088			
No	1	—				
Yes	2.259	0.887, 5.755				
**Local treatment between the ICI courses**			0.100			
No	1					
Yes	0.767	0.260, 2.262				
**Number of metastatic sites (ICI rechallenge)**			0.039			0.020
**> 3**	1	—		1		
**≤ 3**	0.413	0.179, 0.96		0.366	0.157, 0.856	
**Brain metastasis (ICI rechallenge)**			0.283			
No	1	—				
Yes	1.606	0.677, 3.813				
**Elevated LDH level (ICI rechallenge)**			0.275			
No	1	—				
Yes	2.362	0.828, 6.742				
Unknown	1.297	0.486, 3.462				
**Corticosteroids (ICI rechallenge)**			0.195			0.007
No	1	—		1	—	
Yes	2.044	0.692, 6.036		5.395	1.578, 18.44	
**2nd course of ICI (ICI rechallenge)**			0.748			
Rechallenge	1	—				
Escalation	1.350	0.179, 10.16				
Switch	0.598	00.138, 2.595				
**Toxicity (ICI rechallenge)**			0.146			0.025
No	1	—		1	—	
Yes	0.447	0.151, 1.325		0.246	0.072, 0.839	

CI: Confidence interval, ECOG: Eastern cooperative oncology group, ICI: Immune checkpoint inhibitor, LDH: Lactate dehydrogenase, OR: Odds ratio. Only factors which are significant are shown in multivariate analysis

Supplemental Figure S1: Efficacy of the repeated courses of immune checkpoint inhibitors

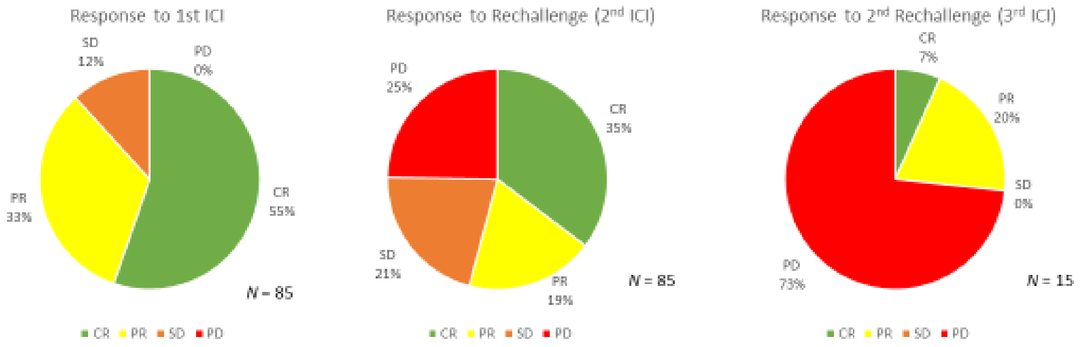

ICI : Immune checkpoint inhibitor; CR : complete response; PR : partial response; SD : stable disease ; PD : progression disease
